# Echinoderms Metabolites: Structure, Functions, and Biomedical Perspectives

**DOI:** 10.3390/md19030125

**Published:** 2021-02-26

**Authors:** Vladimir I. Kalinin

**Affiliations:** G.B. Elyakov Pacific Institute of Bioorganic Chemistry, Far Eastern Branch of the Russian Academy of Sciences, Pr. 100-letya Vladivostoka 159, 690022 Vladivostok, Russia; kalininv@piboc.dvo.ru; Tel.: +7-914-705-08-45

Echinoderms are marine invertebrates belonging to the phylum Echinodermata (from the Ancient Greek words “echinos” (hedgehog) and “derma” (skin)). They have radial symmetry, a unique water vascular (ambulacral) system, and a limestone skeleton, and they include the classes Asteroidea (starfish), Ophiuroidea (brittle stars), Echinoidea (sea urchins), Holothuroidea (sea cucumbers), and Crinoidea (sea lilies). The skeleton of sea cucumbers is reduced by ossicles. Echinoderms have no freshwater or terrestrial representatives and are habitants of all ocean depths. The phylum contains more than 7000 living species [1]. Echinoderms are unique sources of different metabolites having a wide spectrum of biological activities [2]. All echinoderms possess a unique mechanism of decreasing the level of free 5,6-unsaturated sterols in their cell membranes—sulfation of these food sterols [3]. Moreover, sea cucumbers and starfish transform these 5,6-unsaturated sterols into stanols or 7,8-unsaturated sterols, which allows them to synthesize and keep their own 5,6-sterol-dependent membranolytic toxins, namely, triterpene oligoglycosides for sea cucumbers and steroid oligoglycosides for starfish, which have protective significance for the producers [4,5]. Starfish and brittle stars have numerous polyhydroxysteroids and their sulfated and glycosylated derivatives, using them as food emulgators [6]. All echinoderms contain carotenoids [7] and naphthoquinone pigments [8]. The latter are widely presented in sea urchins [9]. The lipid composition of echinoderms is also uncommon and very interesting. For example, they contain cerebrosides [10] and gangliosides [11], which are characteristic of other deuterostomes, including Chordata, Hemichordata, and Tunicata, relatives of echinoderms [12]. Echinoderms contain lectins, glycan-specific glycoproteins with immunity functions for the producers [13], and glycosaminoglycans [14].

In this Special Issue, the echinoderms naphthoquinoids and their analogs are discussed in three articles. The article by Mishchenko et al. concerns the ability of sea urchin pigments (spinochromes)—echinochrome A and its aminated analogues, echinamines A and B—to inhibit different stages of HSV-1 infection in Vero cells and to decrease the virus-induced production of reactive oxygen species. The author found that spinochromes were maximally effective when HSV-1 was pretreated with the tested compounds, revealing the direct effect of spinochromes on the virus particles [15]. Dyshlovoy et al. designed, synthesized, and analyzed a series of 6-S-(1,4-naphthoquinone-2-yl)-D-glucose chimera molecules—novel sugar conjugates of 1,4-naphthoquinone analogs of the sea urchin pigments spinochromes, which were previously known to have anticancer properties. These compounds prevented potential hydrolysis by human glycoside-unspecific enzymes and blocked the Warburg effect—the consumption of sugars by cancer cells that mediates selectivity of their activity against human prostate cancer cells. These substances permeabilize mitochondria membranes, followed by ROS upregulation and release of cytotoxic mitochondrial proteins (AIF and cytochrome C) to the cytoplasm. This leads to the consequent caspase-9 and -3 activation, PARP cleavage, and apoptosis-like cell death [16]. Polonik et al., on the basis of 6,7-substituted 2,5,8-trihydroxy-1,4-naphtoquinone derived from sea urchins, synthesized five new acetyl-O-glucosides of the naphthoquinones. They also synthesized 28 new thiomethylglycosides of 2-hydroxy and 2-methoxy-1,4-naphtoquinones and investigated the cytotoxic activity of 1,4-naphtoquinones (13 compounds) and their O- and S-glycoside derivatives (37 compounds using the MTT method against Neuro-2a mouse neuroblastoma cells). A quantitative structure–activity relationship (QSAR) model of cytotoxic activity of 22 1,4-naphtoquinone derivatives was constructed and tested. The QSAR model was well appropriated for prediction of the activity of 1,4-naphtoquinone derivatives [17]. Hence, the naphthoquinone metabolites and their analogs and derivatives from echinoderms have good medical perspectives as anticancer and antiviral preparations.

Two articles present isolation, structural elucidation, and biosynthetic interpretation of triterpene glycosides from sea cucumbers. The first one concerns the isolation and structural elucidation of thirteen new mono-, di-, and tri-sulfated triterpene glycosides, quadrangularisosides A, A_1_, B, B_1_, B_2_, C, C_1_, D, D_1_–D_4_ and E from the sea cucumber *Colochirus quadrangularis* from Vietnamese shallow waters. The structures of these glycosides were established by 2D NMR spectroscopy and HR-ESI mass spectrometry. The novel carbohydrate moieties of quadrangularisosides D–D_4_, belonging to the group D, and quadrangularisoside E contain three sulfate groups, including one of them occupying an unusual position at C(4) of terminal 3-O-methylglucose residue. Quadrangularisosides A and D_3_ as well as quadrangularisosides A_1_ and D_4_ are characterized by the new aglycones with 25-hydroperoxyl or 24-hydroperoxyl groups in their side chains, respectively. The cytotoxic activities of the isolated compounds were studied. All the compounds were rather strong cytotoxins, but the presence of hydroperoxide groups decreased the activities. Quadrangularisosides A_1_, C, C_1_, and E possessed strong inhibitory activity on colony formation in HT-29 cells. Due to the synergic effects of these glycosides (0.02 µM) and radioactive irradiation (1 Gy), a decreasing number of colonies was detected [18]. The second article concerns the isolation and structural elucidation of six new monosulfated triterpene tetra-, penta- and hexa-osides, namely, kurilosides A_1_, A_2_, C_1_, D, E, and F, as well as the known earlier kuriloside A, with unusual non-holostane aglycones without lactone, from the sea cucumber *Thyonidium* (= *Duasmodactyla*) *kurilensis* (Levin) (Cucumariidae, Dendrochirotida), collected in the Sea of Okhotsk near Onekotan Island from a depth of 100 m. Structures of the glycosides were elucidated by 2D NMR spectroscopy and HR-ESI mass spectrometry. The cytotoxicity of the isolated compounds was studied. The substances have moderate or low activities [19].

The article by Malyarenko et al. concerns the isolation and structural elucidation of four new conjugates, esters of polyhydroxysteroids with long-chain fatty acids from the deep-water Far Eastern starfish *Ceramaster patagonicus*. The structures of the conjugates were elucidated by NMR and ESIMS and all necessary chemical transformations. Very unusual isolated natural products contain 5α-cholestane-3β,6β,15α,16β,26-pentahydroxysteroidal core and differ from each other in terms of the following fatty acid residues: 5′*Z*,11′*Z*-octadecadienoic, 11′*Z*-octadecenoic, 5′*Z*,11′*Z*-eicosadienoic, and 7′*Z*-eicosenoic acids. Only one similar steroid conjugate with a fatty acid was previously isolated from starfish. The action against cancer cells has been studied. Most of the conjugates are moderately cytotoxic, and one conjugate suppressed colony formation and migration of human breast cancer MDA-MB-231 cells and colorectal carcinoma HCT 116 cells [20].

Another article on steroids concerns the use of free sterols as food markers for deep sea sponges, cnidarians, mollusks, crustaceans, and echinoderms. The authors selected echinoids as their model object because the composition of free sterols in sea cucumber and starfish is deviated by their own cytotoxins and dietary sterols significantly transformed by these classes of echinoderms. The identification of sterols was carried out using the GC–MS method. The authors compared the sterol composition of the representatives of three different phyla, namely, Porifera, Cnidaria, and Echinodermata, collected from a deep- and cold-water region on the one hand, and from a shallow tropical area on the other. They found that shallow tropical sponges and cnidarians had plant and zooxanthellae sterols in their tissues, while their deep-sea counterparts displayed phytoplankton and zooplankton sterols. In contrast, echinoids, a class of echinoderms, the most complex phylum along with hemichordates and chordates (deuterostomes), did not show significant differences in their sterol profiles, suggesting that cholesterol synthesis is present in deuterostomes other than protostomes [21].

The final article covers the polymeric metabolites of echinoderms, namely fucosylated chondroitin sulfates (FCSs), PC and HH isolated from the sea cucumbers *Paracaudina chilensis* and *Holothuria hilla*, respectively. The structural characterization of these polysaccharides was performed by chemical transformations and using advanced NMR spectroscopic methods. Both polysaccharides contain a chondroitin core [→3)-β-D-GalNAc (N-acethyl galactosamine)-(1→4)-β-D-GlcA (glucuronic acid)-(1→]_n_, bearing sulfated fucosyl branches at O-3 of every GlcA residue in the chain. These fucosyl residues were different in terms of their character of sulfation: PC contained Fuc2S4S and Fuc4S in a ratio of 2:1, whereas HH possessed Fuc2S4S, Fuc3S4S, and Fuc4S in a ratio of 1.5:1:1. It was also observed that some GalNAc residues in HH contain an unusual disaccharide branch of Fuc4S-(1→2)-Fuc3S4S-(1→ at O-6. Sulfated GalNAc4S6S and GalNAc4S units were found in a ratio of 3:2 for PC and 2:1 for HH, respectively. The polysaccharides revealed significant anticoagulant activity in a clotting time assay, which was caused by the ability of these FCSs to induce the inhibition of thrombin and factor Xa in the presence of anti-thrombin III (ATIII) and with the direct inhibition of thrombin in the absence of any cofactors [22].

The materials published in the Special Issue reflect the real diversity of echinoderm metabolites and cover most of their specific classes and biomedical potential as antioxidant, antiviral, anticancer, and even anticoagulant preparations.
